# Accuracy of genomic-polygenic estimated breeding value for milk yield and fat yield in the Thai multibreed dairy population with five single nucleotide polymorphism sets

**DOI:** 10.5713/ajas.18.0816

**Published:** 2019-02-14

**Authors:** Bodin Wongpom, Skorn Koonawootrittriron, Mauricio A. Elzo, Thanathip Suwanasopee, Danai Jattawa

**Affiliations:** 1 Department of Animal Science, Kasetsart University, Bangkok 10900, Thailand; 2 Department of Animal Sciences, University of Florida, Gainesville, FL 32611-0910, USA

**Keywords:** Accuracy, Cattle, Genome, Imputation, Ranking, Tropics

## Abstract

**Objective:**

The objectives were to compare variance components, genetic parameters, prediction accuracies, and genomic-polygenic estimated breeding value (EBV) rankings for milk yield (MY) and fat yield (FY) in the Thai multibreed dairy population using five single nucleotide polymorphism (SNP) sets from GeneSeek GGP80K chip.

**Methods:**

The dataset contained monthly MY and FY of 8,361 first-lactation cows from 810 farms. Variance components, genetic parameters, and EBV for five SNP sets from the GeneSeek GGP80K chip were obtained using a 2-trait single-step average-information restricted maximum likelihood procedure. The SNP sets were the complete SNP set (all available SNP; SNP100), top 75% set (SNP75), top 50% set (SNP50), top 25% set (SNP25), and top 5% set (SNP5). The 2-trait models included herd-year-season, heterozygosity and age at first calving as fixed effects, and animal additive genetic and residual as random effects.

**Results:**

The estimates of additive genetic variances for MY and FY from SNP subsets were mostly higher than those of the complete set. The SNP25 MY and FY heritability estimates (0.276 and 0.183) were higher than those from SNP75 (0.265 and 0.168), SNP50 (0.275 and 0.179), SNP5 (0.231 and 0.169), and SNP100 (0.251and 0.159). The SNP25 EBV accuracies for MY and FY (39.76% and 33.82%) were higher than for SNP75 (35.01% and 32.60%), SNP50 (39.64% and 33.38%), SNP5 (38.61% and 29.70%), and SNP100 (34.43% and 31.61%). All rank correlations between SNP100 and SNP subsets were above 0.98 for both traits, except for SNP100 and SNP5 (0.93 for MY; 0.92 for FY).

**Conclusion:**

The high SNP25 estimates of genetic variances, heritabilities, EBV accuracies, and rank correlations between SNP100 and SNP25 for MY and FY indicated that genotyping animals with SNP25 dedicated chip would be a suitable to maintain genotyping costs low while speeding up genetic progress for MY and FY in the Thai dairy population.

## INTRODUCTION

Genomic selection refers to selection of animals based on genomic estimated breeding values (GEBV) [1,2]. Current genomic selection procedures utilize a combination of phenotypes, pedigree, and genotype information to improve accuracy of genetic predictions [3]. An important advantage of genomic selection is that it can be implemented to evaluate economically important traits that are difficult to measure such as traits that are expensive to measure in a large number of animals (e.g., feed efficiency), expressed very late-in-life (e.g., length of productive life), and sex-limited traits (e.g., milk yield). In addition, because genomic selection can be performed early in life, it can decrease generation interval, increase intensity of selection, and reduce the cost of proving dairy bulls [1]. Genomic selection is currently widely used for dairy genetic evaluation in many countries. The Thai dairy genomic-polygenic evaluation for economically important traits started in 2015 [4]. Implementation of the genomic-polygenic evaluation system increased EBV accuracies for milk yield (MY) and fat yield (FY) from 5.2% to 7.2% in the Thai multibreed population [4]. Continued genotyping and selection of parents based on genomic-polygenic EBV is expected to accelerate the rate of genetic progress for MY and FY and other evaluated traits in this population.

Prices for high-density genotyping chips are still too high for widespread use in the multibreed dairy cattle population in Thailand. The current option in the Thai dairy population of genotyping sires and highly represented dams in the pedigree with high-density chips and the rest of the cow population with low-density chips may need to remain in place until a suitable cheaper and effective option is found. One such option would be to design low-density chips with small numbers of single nucleotide polymorphism (SNP) that account for a reasonable percentage of the genetic variation for the traits of interest. Use of these low-density assays containing selected SNPs could potentially be as effective as high-density chips for a fraction of the price [5,6]. Determination of an appropriate set of SNP needs to consider the genetic architecture of the traits of interest, population structure, amount of linkage disequilibrium, proportion of ancestors genotyped with high-density SNP genotypes, and genetic relationships between animals used to estimate SNP effects [5]. However, the optimal number of SNP for a low-density chip for dairy cattle in Thailand as well as an appropriate strategy for selecting these SNP have not been studied. Thus, the objectives of this research were to compare estimates of variance components, genetic parameters, accuracies of prediction, and rankings of genomic-polygenic animal EBV for MY and FY in the Thai multibreed dairy population computed using five sets of SNPs from GeneSeek GGP80K chip.

## MATERIALS AND METHODS

### Animals, data and traits

Cattle used in this research were from the Thai multibreed dairy population, a Holstein-upgraded population composed of purebred and crossbred cows, sires, and dams. Breeds represented in Thai dairy cattle are Holstein, Brahman, Jersey, Red Dane, Red Sindhi, Sahiwal, and Thai Native [7]. The largest breed fraction for most animals in the population is Holstein. Although, percentage Holstein per animal ranged from 46.9% to 100%, 91% of cattle in the population was over 75% H plus small percentages of other breeds. The dataset contained monthly test-day MYs and FYs of 8,361 first-lactation cows that calved from 1989 to 2014. These cows were daughters of 1,210 sires and 6,992 dams located in 810 farms across five regions in Thailand (North, Northeastern, Western, Central, and Southern). Traits were 305-d first-lactation MY (kg) and 305-d first-lactation FY (kg). Test-day MYs were measured, and milk samples were taken from each individual cow once a month from calving until drying off. Cow test-day milk samples were sent to a laboratory (Artificial Insemination and Biotechnology Research Centre laboratory, Saraburi province, Thailand) to determine fat percentages. Test-day FYs were computed as the product of test-day MYs times test-day fat percentages. Monthly test-day MYs and FYs were used to compute MY and FY using the test-interval method [8,9].

### Climate, management, and nutrition

The majority of Thailand is hot and humid most of the year. Thailand’s climate is highly influenced by the seasonal monsoon weather characterized by wind pattern changes and heavy precipitation. Seasons are classified as winter (November to February), summer (March to June), and rainy (July to October). Yearly means across regions and seasons range from 17°C to 36°C for temperature, 1,200 to 1,600 mm/yr for rainfall, and 73% to 80% for relative humidity [10]. Cows were housed in open barns and milked twice a day (morning at 5 to 6 am; afternoon at 2 to 3 pm). Farmers used either a bucket system or a pipeline system for milking. Morning and afternoon raw milk was collected in bulk tanks before transporting it to a collection center owned by either dairy cooperative or a private organization. Cows were fed a combination of roughage and concentrate aimed at a rate of 1 kg of feed (16% protein) per 2 kg of milk. Roughage consisted mainly of fresh grasses including Guinea grass (*Penicum maximum*), Ruzi grass (*Brachiaria ruziziensis*), Napier grass (*Pennisetum purpureum*), and Para grass (*Brachiaria mutica*). Other sources of fiber were rice straw and agricultural byproducts (corn cobs, cassava leaves, corn silage).

### Samples and genotypes

Blood or semen samples were collected from 2,661 animals (89 sires and 2,572 dams). Genomic DNA was extracted from semen samples using a GenElute Mammalian Genomic DNA Miniprep Kit (Sigma-Aldrich, St. Louis, MO, USA), whereas a MasterPure DNA Purification Kit (Epicentre Biotechnologies, Madison, WI, USA) was used for blood samples. Concentration and purity of DNA per sample was measured using a Thermo Fisher NanoDrop 2000 spectrophotometer (Thermo Fisher Science Inc., Wilmington, DE, USA). The minimum acceptable DNA concentration was 15 ng/μL with an absorbance ratio of 1.8 at 260/280 nm.

The DNA samples were dried using a Freeze-dry machine for 12 hours Dried DNA samples of 50 μL were airmailed to GeneSeek (Lincoln, NE 68521, USA) for genotyping with GeneSeek Genomic Profiler BeadChips (GGP). Budgetary restrictions determined the type of genotyping chip used for each DNA sample. The GeneSeek Genomic Profiler 80K chip (GGP80K) was used to genotype sires and highly represented cows in the pedigree (n = 139), and lower density chips were used to genotype the remaining cows ([4]; 1,412 with GGP9K; 570 with GGP20K, and 540 with GGP26K). The numbers of SNP markers were 8,590 for the GGP9K, 19,616 for the GGP20K, 25,979 for the GGP26K, and 76,519 for the GGP80K. Nighty five percent of SNP from GGP20K, 47% of SNP from GGP20K, and 54% of SNP from GGP26K were present in GGP80K. Cows genotyped with the three low-density GGP chips were imputed to GGP80K using FImpute 2.2 [11]. The genotype file included SNPs with call rates ≥90% and minor allele frequency ≥0.04 from the 29 autosomes and the X chromosome. After these control restrictions, the genotype file contained 74,148 SNPs per genotyped animal.

### Estimation of variance components for five single nucleotide polymorphism sets

The five SNP sets were defined in terms of the values of SNP variances for MY and FY [12] estimated with program POSTGSF90 (a member of the BLUPF90 Family of Programs) [13]. Firstly, a 2-trait single-step genomic-polygenic model [3,14] was used to estimate variance components for MY and FY using the complete dataset (phenotypes, pedigree, and genotypes) with program AIREMLF90 [15]. Secondly, SNP variances for MY and FY were computed using program POSTGSF90 and ordered from largest to smallest. Then, the following 5 SNP sets (Table 1) were defined: i) complete SNP set (SNP100; 76,519 SNPs); ii) top 75% set (SNP75; 57,390 SNPs); iii) top 50% set (SNP50; 38,260 SNPs); iv) top 25% set (SNP25; 19,130 SNPs); and v) top 5% set (SNP5; 3,826 SNPs).

Estimates of variance components for MY and FY for the five sets of genotypes were computed with AIREMLF90 [15] using the same 2-trait genomic-polygenic model. This model contained herd-year-season, heterozygosity of the cow, and age at first calving as fixed effects, and animal additive genetic and residual as random effects. The mean of the animal additive genetic and the residual effects was assumed to be zero. The variance of the animal additive genetic effects was equal to H ⊗ V_a_, where H is a genomic-polygenic relationship matrix among all animals in the pedigree (with and without genotypes; [16]), and V_a_ is a 2×2 matrix of additive genetic variances and covariances between MY and FY. The variance of residual effects was equal to I ⊗ V*_e_*, where I is an identity matrix, and V*_e_* is a 2×2 matrix of environmental variances and covariances between MY and FY. The convergence criterion for program AIREMLF90 was 10^−12^. The estimates of additive genetic and environmental variances and covariances at convergence for MY and FY were used to compute phenotypic variances and covariances, heritabilities, and genetic, environmental, and phenotypic correlations using the usual expressions. Standard errors of additive genetic and environmental variances and covariances were computed as square roots of diagonal elements of the inverse of the average information matrix computed at convergence. Standard deviations of phenotypic variances and covariances, heritabilities, and genetic, environmental, and phenotypic correlations were computed with a repeated sampling procedure [17] using a set of 5,000 samples.

### Animal estimated breeding values, accuracies, and rankings

Animal EBV for MY and FY for the five SNP sets (SNP5, SNP25, SNP50, SNP75, and SNP100) were computed using the estimates of variance components at convergence for each set of genotypes. The EBV accuracies for each trait were obtained as the correlation between EBV and true breeding values computed using the formula: $\sqrt{1-\frac{PEV}{\hat{\sigma_{a}^{2}}}}\times 100$, where PEV is the prediction error variance (square of the standard error of prediction) and $\hat{\sigma_{a}^{2}}$ is the estimate of the additive genomic-polygenic variance for each SNP set. After computing animal EBV for MY and FY with the five SNP sets, animal EBV were ranked from largest to smallest for MY and from smallest to largest for FY. Subsequently, animal rankings from five SNP sets were compared using the Spearman’s rank correlation procedure of SAS 9.0 (SAS Inst. Inc., Cary, NC, USA).

## RESULTS AND DISCUSSION

### Variances components, heritabilities, and correlations

Estimates of variances and covariances for MY and FY using genomic-polygenic models with five sets of SNP are shown in Table 2 for additive genetic effects, Table 3 for environmental effects, and in Table 4 for phenotypic effects. Similar estimates of additive genetic, environmental, and phenotypic variances and covariances for MY and FY were obtained for the five sets of SNP. Estimates of additive genetic variances ranged from 146,440.00±20,231.00 kg^2^ (SNP5) to 178,510.00±26,620.00 kg^2^ (SNP50) for MY and from 201.54±60.91 kg^2^ (SNP100) to 231.49±57.71 kg^2^ (SNP25) for FY, and additive genetic covariances ranged from 4,041.40±813.25 kg×kg (SNP5) to 4,758.10±984.95 kg×kg (SNP25). Estimates of environmental variances ranged from 467,000.00±22,641.00 kg^2^ (SNP25) to 488,910.00±19,276.00 kg^2^ (SNP5) for MY and from 1,033.30 ±57.18 kg^2^ (SNP25) to 1,065.90±60.37 kg^2^ (SNP100) for FY, and environmental covariances ranged from 14,780.00±945.30 kg×kg (SNP25) to 15,590.00±1,046.00 kg×kg (SNP100). Estimates of phenotypic variances ranged from 635,580.00± 13,307.00 kg^2^ (SNP5) to 650,520.00±13,794.00 kg^2^ (SNP100) for MY and from 1,257.00±33.25 kg^2^ (SNP5) to 1,267.20± 33.61 kg^2^ (SNP75) for FY, and phenotypic covariances ranged from 19,286.00±566.53 kg×kg (SNP5) to 19,678±580.44 kg×kg (SNP75).

Estimates of additive genetic variances and covariances for MY and FY were higher for models with SNP75 (variances: 5.39% for MY, 5.96% for FY; covariances: 8.37%), SNP50 (variances: 9.30% for MY, 12.97% for FY; covariances: 14.83%), SNP25 (variances: 8.43% for MY, 14.86% for FY; covariances: 16.38%) than those estimated using SNP100. However, the model with SNP5 yielded lower estimates of additive genetic variance for MY (−10.34%) and covariance between MY and FY (−1.15%), and higher estimate of additive genetic variance for FY (5.37%) than the model with SNP100. Conversely, environmental variances and covariances for MY and FY were lower for the model with SNP75 (variances: −1.88% for MY, −1.08% for FY; covariance: −2.17%), SNP50 (variances: −3.53% for MY, −2.44% for FY; covariance: −4.11%), SNP25 (variances: −4.10% for MY, −3.06% for FY; covariance: −5.20%) than those estimated using SNP100. The model with SNP5 produced a higher environmental variance for MY (0.40%), but a lower environmental variance for FY (−1.92%) and covariance between MY and FY (−2.19%) than the corresponding estimates from the model with SNP100. Lastly, estimates of phenotypic variances and covariances for MY and FY from models with SNP75, SNP50, and SNP25 were nearly identical (differences were near zero or below one percent) to the corresponding values from model with SNP100, whereas the corresponding differences for the model with SNP5 were all negative and mostly higher than one percent (variances: −2.30% for MY, −0.77% for FY; covariance: −1.97%).

The slightly higher additive genetic variances and covari ances but lower environmental variances and covariances for MY and FY obtained with SNP75, SNP50, and SNP25 than with the complete SNP set indicated that these SNP subsets may have been able to more accurately accounted for MY and FY additive variability in this population than the complete SNP set. This may have occurred because the SNP markers in these subsets were on the average more closely associated with quantitative trait locus (QTL) affecting MY and FY than the complete set of SNP [18].

Heritabilities and additive genetic, environmental, and phenotypic correlations between MY and FY are presented in Table 5. Heritability estimates ranged from 0.231±0.030 (SNP5) to 0.276±0.039 (SNP25) for MY and from 0.159±0.047 (SNP100) to 0.183±0.044 (SNP25) for FY. The SNP25 MY and FY heritability estimates were slightly higher (0.4% to 20%) than those from SNP100, SNP75, SNP50, and SNP5. These differences among heritability estimates across SNP sets may be related to differences in linkage disequilibria between the SNP in each set and QTL affecting MY and FY determined by number of SNP and proximity of SNP in each set to MY and FY QTL [18]. Higher SNP25 heritability estimates for MY and FY indicated that faster selection responses for these traits could be expected with SNP25 than with SNP100, SNP75, SNP50, and SNP5 in the Thai dairy population.

Heritability estimates for MY and FY across the five SNP sets were similar to values estimated in previous studies in the Thai dairy population using various SNP sets (0.19 to 0.26 for MY; 0.15 to 0.18 for FY [4]). Heritabilities for MY in the Thai dairy population were within the range of heritability estimated for MY in various Holstein populations in temperate regions (0.25 to 0.30 [6,19,20]), but somewhat higher than an estimate in Holstein under tropical conditions in Brazil (0.13 [21]). Conversely, heritability estimates for FY were somewhat lower than values obtained for Holstein in temperate regions (0.25 to 0.30 [19,20,22])

Estimates of additive genetic, environmental and pheno typic correlations between MY and FY across the five sets of SNP were virtually identical (Table 5). Correlation estimates between MY and FY ranged from 0.718±0.115 (SNP100) to 0.748±0.087 (SNP25) for additive genetic, from 0.673±0.023 (SNP25) to 0.684±0.023 (SNP100) for environmental, and from 0.682±0.009 (SNP5) to 0.686±0.010 (SNP75) for phenotypic. The positive genetic correlations between MY and FY obtained here were similar to values previously reported for this Thai dairy population (0.66 to 0.79 [4]), and in agreement with estimates for Holstein in other tropical (0.70 to 0.75 [23,24]), and in temperate regions (0.70 to 0.88 [25,26]).

The comparable or slightly higher additive genetic vari ances and heritabilities for MY and FY from SNP25, SNP50, and SNP75 than from SNP100 indicated that selecting a subset of SNP genotypes with the approach used here would be a reasonable alternative to increase the effectiveness of genomic-polygenic evaluation and selection in the Thai dairy population. However, reducing SNP genotypes to 3,826 SNP (SNP5) or 5% of the SNP in the GGP80K chip would yield lower variance component and heritability estimates than with the SNP25, SNP50, and SNP75 subsets, or with the complete SNP set. Further, the highest genetic variance component and heritability estimates for MY and FY obtained with SNP25 indicated that higher EBV prediction accuracies and selection responses for these traits would be achieved using a genomic-polygenic model with SNP25 than with SNP100, SNP75, SNP50, and SNP5.

### Accuracy of genomic-polygenic estimated breeding values and animal rankings with five single nucleotide polymorphism sets

**Figure 1:**
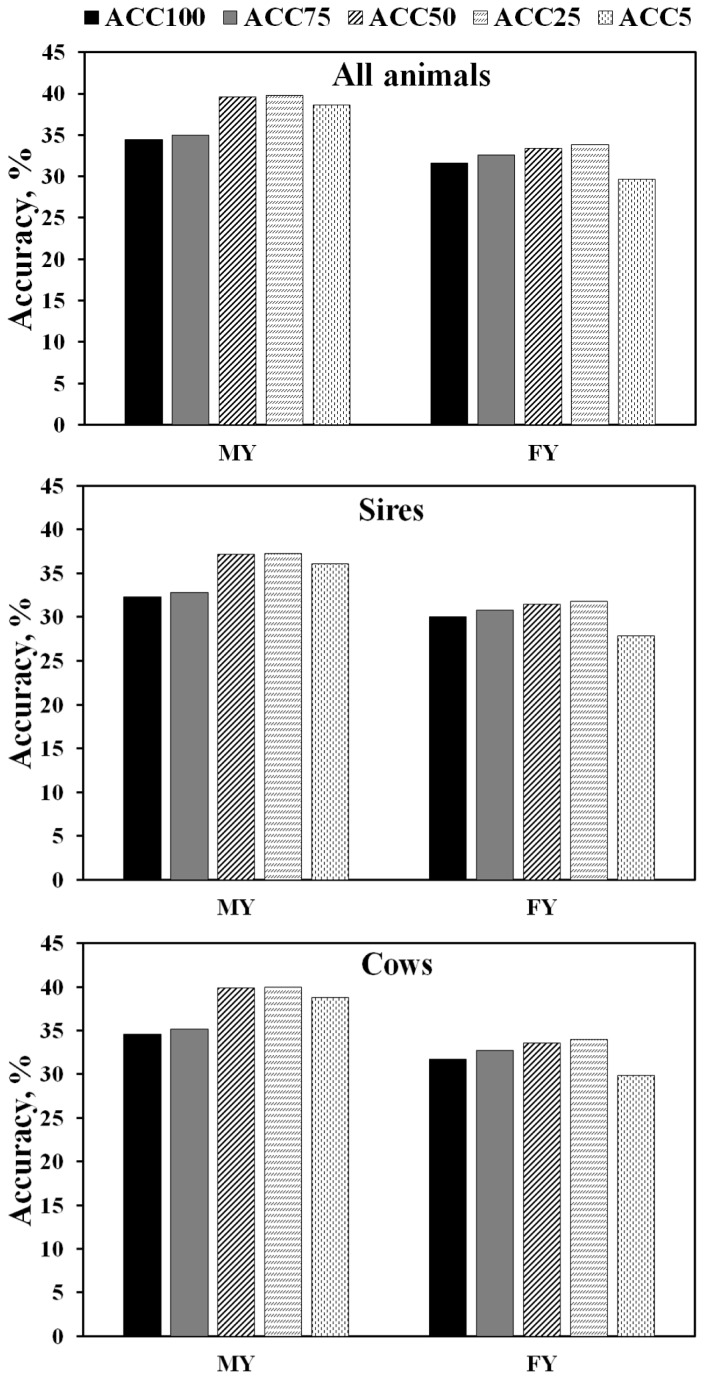
Accuracy of estimated breeding value for MY and FY estimated with five single nucleotide polymorphism sets using genomic-polygenic models. MY, milk yield; FY, fat yield.

The accuracies genomic-polygenic EBV for MY and FY with the five sets of SNP genotypes (SNP100, SNP75, SNP50, SNP25, and SNP5) are shown in Figure 1. The SNP25 had the highest mean EBV accuracy for all animals (39.76% for MY and 33.82% for FY), sires (37.30% for MY and 31.85% for FY), and cows (39.98% for MY and 33.99% for FY). Conversely, SNP100 yielded the lowest mean EBV accuracies for all animals (35.18% for MY and 28.36% for FY), sires (33.12% for MY and 26.94% for FY), and cows (35.36% for MY and 28.49% for FY). Further, the mean EBV accuracies from the four SNP subsets (SNP75, SNP50, SNP25, SNP5) were mostly higher than the mean EBV accuracy from the complete SNP set (SNP100) for all animals, sires, and cows. The percentage superiority of the mean EBV accuracies of SNP75, SNP50, SNP25, and SNP5 over SNP100 for MY were 0.58%, 5.21%, 5.34%, and 4.18% for all animals, 0.54%, 4.92%, 5.01%, and 3.76% for sires, and 0.58%, 5.24%, 5.36%, and 4.21% for cows. Similarly, the percentage superiority of the mean EBV accuracies of SNP75, SNP50, and SNP25 over SNP100 for FY were 0.98%, 1.77%, and 2.21% for all animals, 0.81%, 1.48%, and 1.84% for sires, and 0.99%, 1.80%, and 2.34% for cows. However, the mean EBV accuracies of SNP5 for FY were slightly lower (−1.91% for all animals, −2.18% for sires, and −1.89% for cows) than those of SNP100. The mostly higher mean EBV accuracies of the four SNP subsets were largely due to the higher MY and FY additive genetic variances explained by these SNP subsets than by the complete SNP set. Further, the fact that SNP25 yielded the highest mean EBV accuracy indicated that choosing the top 25% of SNP from GeneSeek GGP80K based on percent of additive genetic variance explained for MY and FY (19,130 SNP) would be a suitable alternative to the complete SNP set for genomic-polygenic evaluation and selection in the Thai dairy multibreed population.

The higher mean EBV accuracies obtained with four GG P80K subsets than with the complete SNP set supported the findings from previous research in dairy [5,27–29] and in beef cattle [30] that SNP subsets can yield comparable or higher levels of EBV accuracy than complete SNP sets while lowering genotyping costs.

Pairwise Spearman rank correlations between MY and FY EBV from of the complete SNP set and each of the four SNP subsets are shown in Table 6. All rank correlations between SNP100 and the four SNP subsets were above 0.98 (p<0.0001) for both traits, except for the correlation between SNP100 and SNP5 (MY, 0.93; FY, 0.92; p<0.0001). Rank correlations between SNP75 and SNP100 and between SNP50 and SNP100 for MY and FY were above 0.99 (p<0.0001), followed closely by rank correlations between SNP25 and SNP100 for MY (0.98; p<0.0001). Rank correlations indicated a high degree of agreement between EBV from genomic-polygenic evaluations with the four SNP subsets and the complete SNP set. The high SNP25 estimates of genetic variances, heritabilities, EBV accuracies, and rank correlations between SNP100 and SNP25 for MY and FY indicated that SNP25 would be expected to produce higher selection responses for MY and FY than any of the other SNP subsets and the complete GeneSeek 80K set. This indicates that a strategy to keep genotyping costs reasonably low while speeding up genetic progress for MY and FY would be to genotype animals in the Thai multibreed dairy population with a dedicated chip constructed with the subset of SNP markers in the SNP25 set. Thai dairy producers could decrease their genotyping costs before the utilization of a dedicated chip likely without reducing their ability to select replacement animals based on genomic-polygenic EBV by utilizing lower-density commercial genotyping chips.

## CONCLUSION

Estimates of additive genetic variances, heritabilities, and EBV accuracies for MY and FY from genomic-polygenic models with SNP subsets were higher than those with complete SNP set. Genomic-polygenic evaluation with SNP25 had the highest estimates of additive genetic variances, heritabilities, and EBV accuracies for MY and FY. Further, genomic-polygenic EBV obtained using SNP subsets and complete SNP set had high rank correlations. Thus, utilization of the SNP25 set would be a suitable alternative to reduce genotyping costs and increase selection response for MY and FY in this dairy population.

## Figures and Tables

**Table 1:** Number of SNP per chromosome and total for the five SNP sets

Chromosomes	SNP sets1)				

	SNP100	SNP75	SNP50	SNP25	SNP5
1	4,512	3,286	2,043	959	166
2	3,914	2,765	1,775	843	157
3	3,566	2,604	1,692	804	165
4	3,398	2,617	1,774	907	165
5	3,528	2,671	1,755	868	163
6	3,399	2,587	1,748	884	163
7	3,209	2,437	1,686	862	176
8	3,248	2,505	1,704	888	170
9	3,085	2,356	1,599	840	152
10	3,007	2,313	1,564	810	186
11	3,094	2,390	1,668	893	225
12	2,662	2,073	1,422	726	156
13	2,468	1,909	1,268	654	123
14	2,495	1,949	1,364	745	180
15	2,579	1,970	1,315	664	167
16	2,471	1,903	1,320	696	143
17	2,229	1,761	1,187	603	140
18	2,056	1,567	1,072	544	118
19	2,012	1,563	1,068	565	128
20	2,228	1,573	1,038	484	84
21	2,220	1,529	990	460	74
22	1,894	1,262	748	325	49
23	1,732	1,210	768	348	71
24	1,936	1,371	888	420	67
25	1,413	984	640	288	38
26	1,618	1,186	762	365	67
27	1,440	1,131	768	387	80
28	1,488	1,180	813	410	69
29	1,596	1,213	821	396	89
X	2,022	1,525	1,000	492	95
Total	76,519	57,390	38,260	19,130	3,826

SNP, single nucleotide polymorphism.

1) The SNP sets were the complete SNP set: SNP100, all available SNP; SNP75, top 75% set; SNP50, top 50% set; SNP25, top 25% set; SNP5, top 5% set.

**Table 2:** Additive genetic variances and covariances for MY and FY estimated with five SNP sets using genomic-polygenic models

Variance component	SNP sets1)									

	SNP100	SE	SNP75	SE	SNP50	SE	SNP25	SE	SNP5	SE
Var (MY, kg^2^)	163,320.00	28,138.00	172,120.00	27,651.00	178,510.00	26,620.00	177,080.00	24,794.00	146,440.00	20,231.00
Cov (MY, FY, kg×kg)	4,088.40	1,094.10	4,430.80	1,081.60	4,694.80	1,049.60	4,758.10	984.95	4,041.40	813.25
Var (FY, kg^2^)	201.54	60.91	213.55	60.71	227.67	60.01	231.49	57.71	212.37	50.40

MY, milk yield; FY, fat yield; SNP, single nucleotide polymorphism; SE, standard error.

1) SNP100, complete SNP set (76,519 SNPs); SNP75, top 75% SNP (57,390 SNPs); SNP50, top 50% SNP (38,260 SNPs); SNP25, top 25% SNP (19,130 SNPs); SNP5 = top 5% SNP (3,826 SNPs).

**Table 3:** Environmental variances and covariances for MY and FY estimated with five SNP sets using genomic-polygenic models

Variance component	SNP sets1)									

	SNP100	SE	SNP75	SE	SNP50	SE	SNP25	SE	SNP5	SE
Var (MY, kg^2^)	486,970.00	25,730.00	477,830.00	25,161.00	469,800.00	24,179.00	467,000.00	22,641.00	488,910.00	19,276.00
Cov (MY, FY, kg×kg)	15,590.00	1,046.00	15,251.00	1,029.70	14,950.00	998.36	14,780.00	945.30	15,248.00	818.88
Var (FY, kg^2^)	1,065.90	60.37	1,054.40	59.95	1,039.90	59.08	1,033.30	57.18	1,045.40	51.53

MY, milk yield; FY, fat yield; SNP, single nucleotide polymorphism; SE, standard error.

1) SNP100, complete SNP set (76,519 SNPs); SNP75, top 75% SNP (57,390 SNPs); SNP50, top 50% SNP (38,260 SNPs); SNP25, top 25% SNP (19,130 SNPs); SNP5, top 5% SNP (3,826 SNPs).

**Table 4:** Phenotypic variances and covariances for MY and FY estimated with five SNP sets using genomic-polygenic models

Variance component	SNP sets1)									

	SNP100	SD2)	SNP75	SD	SNP50	SD	SNP25	SD	SNP5	SD
Var (MY, kg^2^)	650,520.00	13,794.00	650,190.00	13,796.00	648,550.00	13,748.00	644,310.00	13,612.00	635,580.00	13,307.00
Cov (MY, FY, kg×kg)	19,674.00	580.06	19,678.00	580.44	19,641.00	579.37	19,534.00	575.38	19,286.00	566.53
Var (FY, kg^2^)	1,266.70	33.57	1,267.20	33.61	1,266.80	33.62	1,264.00	33.52	1,257.00	33.25

MY, milk yield; FY, fat yield; SNP, single nucleotide polymorphism; SD, standard deviation.

1) SNP100, complete SNP set (76,519 SNPs); SNP75, top 75% SNP (57,390 SNPs); SNP50, top 50% SNP (38,260 SNPs); SNP25, top 25% SNP (19,130 SNPs); SNP5, top 5% SNP (3,826 SNPs).

2) Repeated sampling approach of Meyer and Houle [17].

**Table 5:** Heritabilities and genetic, environmental, and phenotypic correlations between MY and FY estimated with five SNP sets using genomic-polygenic models

Parameter	SNP sets1)									

	SNP100	SD2)	SNP75	SD	SNP50	SD	SNP25	SD	SNP5	SD
Heritability (MY)	0.251	0.041	0.265	0.040	0.275	0.039	0.276	0.036	0.231	0.030
Heritability (FY)	0.159	0.047	0.168	0.047	0.179	0.046	0.183	0.045	0.169	0.039
Genetic correlation (MY, FY)	0.718	0.115	0.736	0.102	0.741	0.094	0.748	0.087	0.674	0.020
Environmental correlation (MY, FY)	0.684	0.023	0.679	0.024	0.676	0.024	0.673	0.023	0.674	0.020
Phenotypic correlation (MY, FY)	0.685	0.010	0.686	0.010	0.685	0.010	0.684	0.010	0.682	0.010

MY, milk yield; FY, fat yield; SNP, single nucleotide polymorphism; SD, standard deviation.

1) SNP100, complete SNP set (76,519 SNPs); SNP75, top 75% SNP (57,390 SNPs); SNP50, top 50% SNP (38,260 SNPs); SNP25, top 25% SNP (19,130 SNPs); SNP5, top 5% SNP (3,826 SNPs).

2) Repeated sampling approach of Meyer and Houle [17].

**Table 6:** Spearman rank correlations between SNP100 and SNP75, SNP100 and SNP50, SNP100 and SNP25, and SNP100 and SNP5 for MY and FY

Trait	Rank correlation1)			

	SNP100, SNP75	SNP100, SNP50	SNP100, SNP25	SNP100, SNP5
MY	0.997	0.991	0.976	0.926
FY	0.997	0.990	0.976	0.919

SNP, single nucleotide polymorphism; MY, milk yield; FY, fat yield.

1) SNP100, complete SNP set (76,519 SNPs); SNP75, top 75% SNP (57,390 SNPs); SNP50, top 50% SNP (38,260 SNPs); SNP25, top 25% SNP (19,130 SNPs); SNP5, top 5% SNP (3,826 SNPs);

All Spearman rank correlations were significant (p<0.0001).
